# Ctrl + Alt + Inner Speech: A Verbal–Cognitive Scaffold (VCS) Model of Pathways to Computational Thinking

**DOI:** 10.3390/jintelligence13120156

**Published:** 2025-12-02

**Authors:** Daisuke Akiba

**Affiliations:** Queens College and the Graduate Center, The City University of New York, Queens, NY 11367, USA; dakiba@qc.cuny.edu

**Keywords:** computational thinking, executive function, inner speech, metacognition, problem solving, self-regulation, verbal mediation

## Abstract

This theoretical paper introduces the Verbal–Cognitive Scaffold (VCS) Model, a cognitively inclusive framework which proposes the cognitive architectures underlying computational thinking (CT). Moving beyond monolithic theories of cognition (e.g., executive-function and metacognitive control models), the VCS Model posits inner speech (InSp) as the predominant cognitive pathway supporting CT operations in neurotypical populations. Synthesizing interdisciplinary scholarship across cognitive science, computational theory, neurodiversity research, and others, this framework articulates distinct mechanisms through which InSp supports CT. The model specifies four primary pathways linking InSp to CT components: verbal working memory supporting decomposition, symbolic representation facilitating pattern recognition and abstraction, sequential processing enabling algorithmic thinking, and dialogic self-questioning enhancing debugging processes. Crucially, the model posits these verbally mediated pathways as modal rather than universal. Although non-verbal architectures are acknowledged as possible alternative routes, their precise mechanisms remain underspecified in the existing literature and, therefore, are not the focus of the current theoretical exploration. Given this context, this manuscript focuses on the well-documented verbal support provided by InSp. The VCS Model’s theoretical contributions include the following: (1) specification of nuanced cognitive support systems where distinct InSp functions selectively enable particular CT operations; (2) generation of empirically testable predictions regarding aptitude–pathway interactions in computational training and performance; and (3) compatibility with future empirical efforts to inquire into neurodivergent strategies that may diverge from verbal architectures, while acknowledging that these alternatives remain underexplored. Individual variations in InSp phenomenology are theorized to predict distinctive patterns of CT engagement. This comprehensive framework, thus, elaborates and extends existing verbal mediation theories by specifying how InSp supports and enables CT, while laying the groundwork for possible future inquiry into alternative, non-verbal cognitive pathways.

## 1. Introduction

Inner speech (InSp), the silent act of “talking to ourselves” in our minds, is a fundamental cognitive process supporting higher-level functions (Reinboth and Farkaš 2022). From planning daily schedules to solving complex problems, InSp provides cognitive support that helps structure and organize our thinking while regulating our behavior. Concurrently, computational thinking (CT), originally conceptualized in computer science as a tool to “translate” problem-solving processes into systematic, automatable steps (Wing 2006), has gained prominence for its emphasis on sequentially organized problem-solving approaches consisting of such cornerstones as *decomposition*, *pattern recognition*, *abstraction*, and *algorithmic thinking* (Anderson 2016). CT, in the recent years, has garnered attention not only in computer science but also in cognitive science, educational psychology, medicine, and neuroscience for its potential to explain domain-general problem-solving strategies (Akiba and Hirano 2025; Li et al. 2020; Shute et al. 2017). A growing body of scholarship now also recognizes debugging, the process of iteratively monitoring, evaluating, and refining problem-solving strategies, as an essential component of CT, especially in educational and training contexts (Shute et al. 2017; Zhang and Nouri 2019). Despite their apparent similarities in such features as reliance on abstract representation, sequential processing, and self-regulation, the relationship between these two cognitive phenomena remains surprisingly unexplored. This manuscript proposes a theoretical framework, the Verbal–Cognitive Scaffold (VCS) Model, intended to advance novel conceptual connections between InSp and CT.

This paper advances new theoretical lenses for understanding the cognitive mechanisms underlying CT. Classic frameworks such as Baddeley’s model of working memory (Baddeley 1992, 2010) and Fodor’s account of modularity (Fodor 1983) illustrate how conceptual synthesis can guide and structure future empirical inquiry. In keeping with the “hypothesis and theory” framing, the present manuscript develops a conceptual model synthesizing existing research on InSp and CT rather than reporting new data. Although the model positions InSp as the predominant support for CT among neurotypical populations, it adopts a pluralistic stance that recognizes the potential viability of alternative, non-verbal cognitive pathways. This commitment to theoretical inclusivity reconceptualizes computational cognition as a multiply realizable process, meaning one that can be implemented through different cognitive routes. Verbal mediation may therefore be typical but not exclusive within this broader framework.

Recent theoretical and empirical developments across multiple disciplines converge to suggest previously unexplored connections between InSp and computational cognition. The reconceptualization of CT as fundamentally cognitive rather than merely technical (Li et al. 2020; Wing 2006, 2016) opens new theoretical space for examining its underlying mechanisms. Concurrently, advances in InSp research reveal its multifaceted role in executive functioning, working memory, and metacognitive regulation (Fama et al. 2024; Grandchamp et al. 2019), while emerging work in cognitive robotics demonstrates how verbal processes support computational operations through categorization, abstraction, and symbolic representation (Giorgi et al. 2023; Chella et al. 2020). These parallel developments invite theoretical synthesis; however, no existing framework appears to explicitly theorize the cognitive mechanisms explicitly linking InSp to CT processes. This theoretical gap may be especially notable, given the ostensibly clear parallels between InSp functions and CT operations, as described in detail in later sections. Both domains involve symbolic manipulation, sequential processing, recursive self-monitoring, and hierarchical problem decomposition (Alderson-Day and Fernyhough 2015; Anderson 2016).

The lack of theoretical integration between InSp and CT remains a major obstacle to understanding how humans develop and apply computational reasoning. The shared characteristics of the two domains suggest a dynamic interplay, with InSp functioning as a primary cognitive support for CT processes, as elaborated in Section 2. While the proposed Verbal–Cognitive Scaffold (VCS) framework emphasizes verbal–cognitive mediation as the dominant pathway, it also recognizes cognition’s plural forms, ready to accommodate non-verbal routes such as visual, motoric, and affective representations that may guide CT through alternative mechanisms. To explore these relationships, this novel framework is guided by the following overarching question: *Which specific cognitive functions of InSp support the development and execution of CT processes?*

Four secondary guiding questions further operationalize this inquiry:

Guiding Question 1. *What cognitive mechanisms mediate the link between InSp and the four cornerstone components of CT—decomposition, pattern recognition, abstraction, and algorithmic reasoning—as well as debugging?*

Guiding Question 2. *Which functions of InSp (e.g., self-regulation, inner rehearsal, dialogic reflection) align most closely with specific CT processes?*

Guiding Question 3. *How do atypical cognitive profiles (e.g., neurodiverse populations) inform our understanding of alternative, non-verbal pathways into CT?*

Guiding Question 4. *How can understanding InSp’s role in CT inform interventions that foreground verbal processes while acknowledging cognitively diverse routes to CT?*

## 2. Theoretical Foundations and Key Constructs

To understand how InSp may support CT, it would first be necessary to establish the theoretical lens and define the key constructs involved. This section introduces the Verbal–cognitive Scaffold (VCS) Model, an integral theoretical framework being explored, and then examines CT and InSp through this cognitive perspective. Upon establishing these foundations, we can explore their intricate relationships in subsequent sections.

### 2.1. Theoretical Scope and Methodological Approach

This subsection establishes how InSp functions as a representational and regulatory system, forming the foundation for complex cognition. The paper adopts a theoretical synthesis methodology that integrates insights from cognitive psychology, computer science, educational psychology, and research on neurodiversity. This interdisciplinary approach follows established precedents for conceptual contribution (e.g., Newell 1990; Marr 1982), in which theoretical frameworks provide essential organizing principles prior to empirical validation. The propositions advanced here draw on extensive empirical findings from related domains; however, the direct integration of InSp and CT represents an uncharted theoretical connection that opens a new avenue for future empirical investigation.

The VCS Model’s theoretical novelty emerges through its synthesis of previously disconnected cognitive dimensions. While existing frameworks have examined executive function (Diamond 2013) or metacognition (Flavell 1979) in CT contexts, none seems to have theorized the specific mediating role of InSp’s multidimensional architecture. This gap is particularly striking given recent developments in cognitive robotics (Chella et al. 2020) and phenomenological approaches to inner experience (Grandchamp et al. 2019), which converge on the centrality of verbal processes in computational reasoning. In short, this manuscript focuses on the well-documented verbal structure provided by InSp vis-à-vis CT. In this manuscript, CT is defined as a domain-general cognitive framework involving structured problem-solving operations such as decomposition, abstraction, pattern recognition, and algorithmic reasoning (Anderson 2016; Wing 2006). CT is therefore differentiated from computing, the external, technical execution of those operations in code or machines, and from computational reasoning, the real-time cognitive enactment of CT strategies inside the thinker.

The VCS Model theorizes how verbal–cognitive processes, chiefly InSp, support the execution of CT and, by extension, inform pedagogical approaches that reach beyond purely technical instruction. Although certain InSp mechanisms may operate partly at sub-personal levels (Alderson-Day and Fernyhough 2015; Grandchamp et al. 2019), they remain accessible to introspection and can therefore be analyzed at the personal level. Consistent with Wing’s original conception of CT as deliberate mental activity (Wing 2006) and with Grover and Pea’s educational framing (Grover and Pea 2013), the present framework keeps its focus on conscious, algorithmic manipulation of mental representations. Fully sub-personal definitions of computation, such as Bechtel’s mechanistic account (Bechtel 2019), offer valuable insights but lie outside the present scope, which is limited to subjectively accessible processes that can be leveraged in instructional contexts.

To prevent possible “terminological slippage,” thus, this manuscript follows a consistent hierarchy: CT refers to the overarching framework; computational reasoning (and its derivatives, such as computational problem solving) signals moment-to-moment implementation of CT operations; and computing is used only for machine execution or code. This general usage avoids the conflation found in prior literature and keeps the focus on how InSp enables the cognitive, rather than purely technical, dimensions of CT.

### 2.2. The Verbal–Cognitive Scaffold (VCS) Model: A Proposed Framework

As initial steps toward theoretical development, this paper proposes the VCS Model, which posits that InSp serves as the primary cognitive processes supporting complex thought in neurotypical individuals. It traces theoretical developments from classic verbal-mediation accounts to contemporary models that emphasize InSp’s active structuring role in cognition. This model advances beyond existing frameworks in three ways. First, unlike traditional verbal mediation theories (Vygotsky 1962), the VCS Model specifies the mechanisms through which InSp structures complex cognition. Second, while metacognitive control models (Nelson and Narens 1990) focus on monitoring processes, the VCS Model emphasizes InSp as an active structuring agent shaping cognitive operations in real-time. Finally, the model integrates findings from cognitive science, neuroscience, education, to computing research to provide a unified account of how language supports thought, bridging previously disconnected research domains.

Unlike broader executive function or metacognitive models, which describe general self-regulatory control, the proposed VCS Model offers a novel mechanistic account of how InSp underpins CT. It specifies, for the first time to the author’s knowledge, the functional routes—such as phonological rehearsal and verbal abstraction—through which linguistic cognition operationalizes each CT component. This represents a previously unexplored theoretical space in the CT literature, moving beyond descriptive executive function-based accounts to establish a distinct, empirically testable framework for understanding how language-based cognition structures complex thought.

### 2.3. CT from a Cognitive Perspective

In the VCS Model, CT represents a complex cognitive process that may be particularly dependent on verbal support. Although CT originated in computer science, it has gained widespread attention in technology education, coding instruction and PK-12 education, where it is often framed as a set of core problem-solving approaches (Anderson 2016). CT has been conceptualized in vastly different ways across disciplines. Denning and Tedre (2019) emphasize that CT is not merely a finite set of strategies but a broad epistemological framework encompassing an overarching way of knowing, reasoning, and engaging with complexity through computational principles. While acknowledging this broader conception, consistent with most psychological and educational research (Grover and Pea 2013; Shute et al. 2017; Wing 2006), the model adopts a pragmatically bounded approach centering on four key components (i.e., decomposition, pattern recognition, abstraction, and algorithmic thinking), plus debugging, as a critical metacognitive overlay that captures the iterative, error-correction dynamics crucial to CT practice. These five dimensions have been most systematically operationalized in cognitive and pedagogical studies on CT to date.

The first of these processes under explicit analysis in this model, *decomposition*, involves breaking complex problems into smaller, manageable parts. Cognitively, this process primarily engages chunking mechanisms and hierarchical representation in working memory, relying on executive functions to prioritize subgoals and sequence tasks logically (Miyake et al. 2000). When confronted with complex problems, individuals systematically identify component elements and their relationships by strategically dividing the problem space, allowing cognitive load reduction as subcomponents can be stored, manipulated, or solved in parallel. This often involves building mental “problem trees” or recursive structures that encapsulate intermediate steps, a cognitive maneuver well documented in expert problem solvers across domains (Sweller 1988).

*Pattern recognition* constitutes the second cornerstone process, involving the identification of similarities within and between problems (Robledo-Castro et al. 2023). This process draws on categorization abilities and inductive reasoning, allowing individuals to leverage previously encountered solutions when facing new problems. Cognitively, pattern recognition reflects our ability to extract regularities from experience and apply them to novel situations, which is a fundamental aspect of human learning and problem-solving. Crucially, pattern recognition operates on multiple levels: identifying recurring structures within single problem spaces and recognizing cross-problem patterns. This flexibility allows pattern recognition to support both localized insight and generalized strategy formation. The process engages schema activation and analogical transfer, where past experiences are retrieved and mapped onto new tasks based on structural or surface similarities (Gick and Holyoak 1980), reducing cognitive load and enabling strategic reuse of prior knowledge.

*Abstraction*, the third cornerstone, involves focusing on relevant information while filtering irrelevant details (Wing 2006). Unlike pattern recognition’s identification of similarities, abstraction entails isolating underlying principles that generalize beyond context. This higher-order function requires selective attention and conceptual representation, enabling development of mental models that capture essential problem features while disregarding superfluous aspects. As Buckner (2018) suggests, abstraction involves forming internal models that enable generalization and prediction across contexts and domains. This capacity enables flexible representational thinking, supporting transitions between specific cases and higher-order generalizations, allowing individuals to connect concrete instances to general principles—a capability central to transferable problem solving.

The fourth cornerstone, *algorithmic thinking,* involves articulation of step-by-step procedures for solving problems, which can be followed by humans or machines (Lodi and Martini 2021). This process engages sequential reasoning and conditional logic, requiring individuals to plan and articulate solution pathways. Cognitively, algorithmic thinking reflects our capacity for procedural thinking and logical inference (i.e., abilities that extend far beyond programming contexts to everyday problem-solving).

Additionally, *debugging*, the iterative process of identifying and resolving errors, functions as a critical metacognitive overlay spanning all four CT cornerstones rather than a fifth component. This process involves monitoring, error detection, hypothesis generation, and cognitive flexibility. As an overlay, debugging enables the self-corrective cycles that characterize computational reasoning and underscore personal-level agency in problem-solving. Metacognitive monitoring within CT involves evaluating the accuracy and reliability of one’s own reasoning processes, which is a capacity closely tied to self-regulated cognition. As Fischer and Fleming (2024) emphasize, metacognitive insight (i.e., the awareness that one’s beliefs or judgments may be subject to error) plays a critical role in adaptive evidence evaluation and in reducing cognitive rigidity across domains. This aligns with the VCS Model’s view of debugging as a metacognitive loop that enhances openness to correction and iterative refinement of thought.

From a cognitive science perspective, thus, CT involves domain-general executive functions applied to specific problem-solving contexts. Rather than representing a specialized skill limited to computing contexts, CT reflects fundamental aspects of human cognition applied in systematic ways. The selection of these key aspects of CT (i.e., decomposition, pattern recognition, abstraction, algorithmic thinking, and debugging) reflects both theoretical considerations and empirical findings regarding the cognitive operations most central to computational problem-solving in the current framework (Grover and Pea 2013; Wing 2006). While related cognitive processes such as analogical reasoning, cognitive control, and hypothetical thinking certainly contribute to CT, the key CT processes under scrutiny in the proposed model represent a commonly articulated set of CT operations that distinguish computational approaches from general problem-solving strategies (Brennan and Resnick 2012; Shute et al. 2017).

### 2.4. InSp as a Cognitive Mechanism

InSp, phenomenologically experienced as silent self-talk, remains a theoretically contested construct requiring precise conceptualization. Following recent debates (Gregory 2020; Fernyhough 2017; Quilty-Dunn et al. 2023), the present model adopts a functional–mechanistic view encompassing both conscious verbal experience and sub-personal linguistic processing. Unlike the Language of Thought hypothesis, which treats internal linguistic representations as thought itself, the VCS Model posits that InSp *guides*—but does not constitute—computational reasoning, emphasizing a metacognitive rather than constitutive role for verbal mediation. This distinction emphasizes the model’s metacognitive rather than constitutive conceptualization of verbal mediation. Additionally, this stance aligns with articulatory suppression findings and contemporary views of InSp as a multidimensional construct (Grandchamp et al. 2019; Alderson-Day and Fernyhough 2015). This functional stance thus permits modeling both deliberative and automatic forms of verbal support across CT tasks.

From a cognitive perspective, InSp involves more than simply “hearing” words internally, as it also functions as an active process that supports various cognitive operations through verbal self-regulation and representation (Roelofs 2020). One of its most well-established roles may be in working memory. As proposed in Baddeley’s (1992) influential model, verbal rehearsal can maintain memory traces and improve recall, as long as articulation is sustained (Alderson-Day and Fernyhough 2015). The phonological loop component of working memory enables the maintenance of verbal information through subvocal repetition, supporting a range of tasks from recalling phone numbers to following complex instructions. This connection between InSp and working memory offers a foundation for understanding how verbal self-regulation might support computational processes that involve managing multiple problem representations (Borghi 2020). Beyond its role in working memory, InSp also exhibits several functional dimensions that differentially support cognitive operations, including *dialogicity*, *condensation*, *intentionality*, and *phenomenological richness* (Grandchamp et al. 2019; McCarthy-Jones and Fernyhough 2011).

Building on this functional and dimensional foundation, InSp appears to exhibit several characteristics that make it particularly well suited for complex problem solving. Its dialogic quality enables individuals to simulate internal conversations, engage in question–answer sequences, and adopt multiple perspectives on a problem (Emerson and Miyake 2003). This dialogicity supports processes such as hypothesis testing and the evaluation of alternative solution paths. The condensed form of InSp enhances cognitive efficiency, allowing for rapid cycling through internal representations. Perhaps most critically for CT, InSp plays a regulatory role by guiding behavior and sustaining goal-directed activity. As Fernyhough and Fradley (2005) demonstrated, this self-regulatory function is central to executive functioning (Alderson-Day and Fernyhough 2015; Borghi 2020). Through verbal self-guidance, InSp enables individuals to plan, monitor, and adjust their thinking, which all represent functions that align closely with the demands of computational reasoning.

InSp also varies substantially across individuals in both frequency and form (McCarthy-Jones and Fernyhough 2011; Nedergaard and Lupyan 2024). Some experience it as a rich internal dialog, while others report more condensed or fragmented versions. Yet others describe little or no InSp at all, relying instead on other modalities, such as visual imagery or unsymbolized thought, to guide cognition. These differences suggest that individuals may recruit distinct cognitive tools when approaching problem-solving tasks, including those associated with CT. Such variation also extends across neurocognitive profiles. For example, individuals with autism spectrum conditions (ASC) often report reduced or qualitatively different experiences of InSp, yet they may display strong computational reasoning skills (Williams et al. 2012). Similarly, individuals with anendophasia (i.e., those who report an absence of InSp) have shown intact reasoning abilities.

These findings raise important theoretical questions about whether InSp is *necessary* for CT, or whether it represents one *common-but-not-exclusive* pathway among multiple forms of computational cognition. Section 3 examines how specific InSp features map onto CT components, and Section 4 addresses the implications of cognitive variability, proposing that verbal mediation may be the prevailing—but not the only—route to CT.

### 2.5. Theoretical Integration: Connecting the Constructs

Prior theoretical work has explored various aspects of CT from pedagogical, developmental, and assessment perspectives (Grover and Pea 2013; Zhang and Nouri 2019). However, these frameworks primarily address what CT is and how it develops, rather than the underlying cognitive mechanisms that enable these processes to transpire. Similarly, while executive function research has identified correlations among working memory, cognitive flexibility, and CT outcomes (Zhang et al. 2025), the specific cognitive operations linking these relationships remain underexplored. The proposed VCS Model responds to this limitation by envisioning InSP as the core cognitive mechanism that enables and supports CT operations through specific, identifiable pathways. It posits that InSp provides critical cognitive support for the complex problem-solving processes involved in CT through specific mechanisms that align with each component of CT.

**Figure 1:**
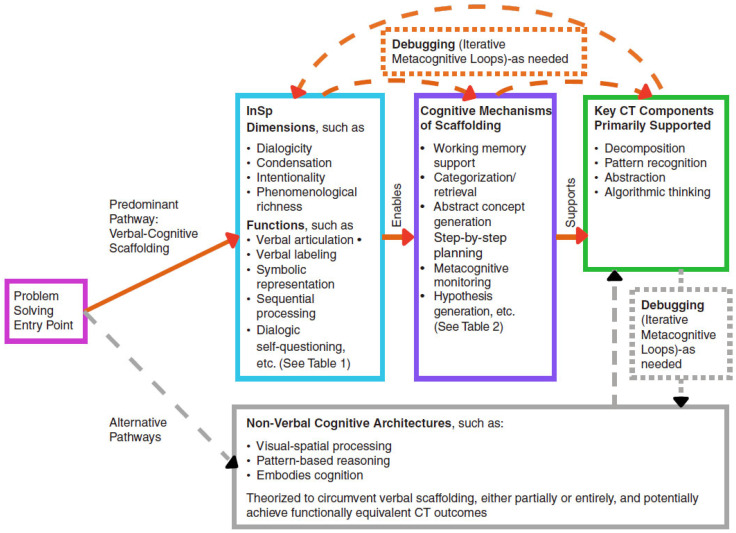
The Verbal–Cognitive Scaffold (VCS) Model illustrating pathways to Computational Thinking (CT). The figure depicts the primary verbal–cognitive pathway through which InSp facilitates CT. Solid orange arrows trace the predominant pathway, showing how InSp dimensions and functions (blue box) enable cognitive mechanisms of scaffolding (purple box), which in turn support the four key CT components (green box). Dashed orange arcs represent debugging as an iterative metacognitive loop that can recur across components. The lower route in gray acknowledge alternative, theorized non-verbal cognitive architectures (e.g., visual–spatial or embodied reasoning) that may achieve similar CT outcomes but are not modeled in detail here, as empirical research on these pathways remains limited.

The theoretical constructs delineated above require a systematic mapping to clarify their interconnected architecture. Table 1 provides that first map, laying out the dynamic interplay among phenomenological dimensions, functional mechanisms, and CT outcomes. To facilitate conceptual clarity, Table 1 is presented in two panels. Panel A outlines the phenomenological dimensions of InSp and their cognitive functions, whereas Panel B summarizes the corresponding components of CT. This dual-panel structure is intended to highlight the model’s parallel organization and to allow readers to view both conceptual layers concurrently, aligning with the architecture visualized in Figure 1 and extended in Table 2.

These panels supply the conceptual building blocks for understanding how verbal–cognitive structure links InSp to CT, visualized in Figure 1 below and mapped to mechanisms in Table 2. Although recent Language of Thought architectures (Quilty-Dunn et al. 2023) treat internal linguistic representations as thought’s medium, the VCS Model advances a distinct claim: InSp promotes rather than constitutes computational reasoning, serving primarily a metacognitive role. Figure 1 illustrates how these components connect through verbal–cognitive scaffolding and propose testable links between InSp features and CT outcomes (e.g., dialogic InSp may enhance metacognitive monitoring during debugging, while condensation supports working-memory efficiency during algorithmic planning). These hypotheses frame the model’s mechanistic interpretation of CT processes and are schematically represented in Figure 1 below.

Empirically, substantial evidence supports this theoretical stance. Systematic reviews show think-alouds illuminate and enhance CT moves (Pan et al. 2024); robot-programming studies link richer spoken self-explanations to higher CT gains (Gong et al. 2025); and prompted dialog improves troubleshooting in debugging (DeLiema et al. 2025). Even in early-childhood settings, educators’ verbal prompts foster effective CT engagement, particularly in sequencing and abstraction (Murcia et al. 2024). The VCS Model, thus, situates that transition in InSp as the personal-level workspace where informal descriptions are processed, rehearsed and refined before formal representation is produced; hence InSp is treated as a modal scaffold for CT, not a necessary or constitutive medium. Methodologically, Pan et al. (2023) detail safeguards for collecting authentic think-aloud data in CT research. Given this substantial empirical foundation demonstrating verbal scaffolding of CT, it should be noted that the VCS Model’s primary focus on InSp-mediated pathways reflects the current state of empirical knowledge rather than theoretical limitations or bias.

As shown in Figure 1, the VCS Model conceptualizes CT as an extension of InSp-driven cognition. InSp provides the representational and metacognitive resources necessary for the four key components of CT and, when needed, powers iterative refinement through debugging. By emphasizing these processes, the model frames CT not merely as a technical skill set but, instead, as a structured expression of verbal mediation and self-regulated thought. The model also acknowledges that some individuals may rely on nonverbal routes, such as visual–spatial or embodied reasoning, to achieve comparable outcomes, although these pathways are not elaborated here due to the limited empirical evidence currently available on such processes.

## 3. Cognitive Mechanisms of InSp in CT

The proposed relationships between InSp and CT operate through multiple cognitive processes, each tailored to specific CT components. These pathways reflect the multidimensional nature of InSp, with functional dimensions mapped onto corresponding CT operations. This section examines each function in detail to build an integrated model of verbal–cognitive scaffolding. Although Table 2 summarizes these cognitive mechanisms, the narrative below provides explanatory depth. Rather than suggesting that language universally determines cognition, the model treats InSp as a domain-specific support, especially effective, though not exclusive, for computational problem-solving (cf. Fedorenko and Varley 2016, on language–thought dissociation).

Table 2 presents individual mechanisms; however, InSp functions interact dynamically to support CT. With this overview in place, we now examine how each function relates to CT components. Before proceeding, however, it is worth addressing why the VCS Model focuses specifically on inner rather than external speech.

InSp operates with significantly greater speed and efficiency than overt verbalization, allowing rapid iterative processing essential for complex problem-solving (Fernyhough and Fradley 2005). Its condensed nature enables maintenance of multiple problem representations without overwhelming working memory capacity. Additionally, InSp seamlessly integrates with visual-spatial processing, whereas external verbalization may disrupt such reasoning (Alderson-Day and Fernyhough 2015). These properties make InSp particularly suited for CT’s cognitive demands. While external verbalization has pedagogical value, InSp provides the cognitive efficiency necessary to support real-time CT processes.

### 3.1. InSp and Cognitive Processes in Problem Decomposition

Decomposition (i.e., the breaking down of complex problems into manageable components) represents a foundational aspect of CT (Yadav et al. 2014). Several interrelated InSp mechanisms involving working memory and verbal representation provide critical cognitive support for decomposition. InSp enables individuals to verbally articulate problem components, essentially “talking through” the problem structure (Alderson-Day and Fernyhough 2015), utilizing the phonological loop to maintain multiple problem representations in working memory (Emerson and Miyake 2003). According to this principle, by labeling and rehearsing the key problem components verbally, individuals reduce distractions and, thus, cognitive load, during decomposition tasks, freeing attentional resources for deeper analysis. The sequential nature of speech (whether external or internal) provides a natural *platform* for analyzing problem elements in an orderly fashion (Fama et al. 2024). Unlike visual processing, which often involves parallel processing of multiple elements, verbal processing unfolds temporally, allowing systematic consideration of problem components (Scott and Perrachione 2019). It would therefore be reasonable to propose that fluency in this sequential verbal processing promotes comprehensive problem analysis without overlooking crucial elements, thereby enhancing one’s ability to engage in decomposition.

Furthermore, verbal self-guidance helps establish relationships among decomposed components through explicit articulation. When a individuals uses InSp to describe how components relate to one another, they are essentially constructing a hierarchical mental representation of the problem (Fernyhough and Fradley 2005). These verbally mediated hierarchical representations would naturally support the construction of “problem models” in working memory that preserve structural relationships. The representational role of InSp may be particularly important for novices in CT, who have yet to develop fluency in CT. For those learning CT, verbal mediation provides a familiar cognitive guidance that bridges everyday reasoning and more specialized computational representations (Angeli and Georgiou 2023). As expertise develops, however, this verbal scaffolding may become less necessary for familiar aspects of CT, though it likely remains important for unfamiliar or particularly complex problems.

### 3.2. InSp and Cognitive Mechanisms in Pattern Recognition

Pattern recognition in CT involves identifying similarities between and within problems, allowing the application of known solutions to new contexts. InSp supports this process through cognitive mechanisms involved in categorization, comparison, and memory retrieval, helping individuals identify, organize, and extract recurring features across stimuli in order to recognize meaningful patterns and connect them to prior knowledge (Fernyhough and Borghi 2023). The linguistic nature of InSp provides powerful tools for categorization through verbal labeling. When computational thinkers encounter potential patterns, InSp allows them to assign verbal labels that facilitate categorization and later retrieval (Robledo-Castro et al. 2023). These verbal categories support the formation and application of computational patterns by organizing experiences into meaningful groups that transcend surface-level differences.

Beyond simple labeling, InSp supports comparative analysis between current problems and previously encountered situations. Through verbal formulation, it could be presumed that individuals can explicitly articulate similarities and differences, making implicit patterns explicit through language. This process of verbalization shall help reveal structural similarities that might otherwise remain unnoticed, particularly when surface features differ dramatically. InSp also appears to play a crucial role in memory retrieval during pattern recognition. Verbal cues generated through InSp can activate relevant prior knowledge and experiences stored in long-term memory. When a computational thinker verbally notes that a current problem “looks like” a previously solved problem, this verbal association serves as a retrieval cue that can activate relevant solution strategies from memory.

Additionally, InSp should enable the explicit formulation of pattern rules, supporting their conscious application to new instances. By articulating patterns verbally, computational thinkers transform implicit recognition into explicit rules that can be systematically applied and taught to others. This transformation from implicit pattern detection to explicit rule formulation represents a key aspect of CT that appears heavily supported by InSp. When synthesized, these verbal-labeling mechanisms yield testable predictions; for example, blocking overt or covert labeling via articulatory suppression should impair pattern retrieval, providing an experimental lever for future studies.

### 3.3. InSp and Cognitive Functions in Abstraction

Abstraction, the process of prioritizing essential information while filtering out less relevant aspects, constitutes the most cognitively demanding aspect of CT (Qian and Choi 2023). InSp supports abstraction through symbolic representation and generalization. Buckner’s (2018) model-based prediction framework emphasize that abstraction involves constructing generalizable mental models for cross-domain prediction, not merely stripping away details. The VCS Model complements this by suggesting that InSp enables model construction through verbal labeling and sequencing, facilitating the recognition, especially in educational and reflective settings. The symbolic nature of language makes InSp an ideal medium for abstract representation (Kompa and Mueller 2020). Unlike perceptual representations tied to concrete instances, linguistic symbols capture general categories and concepts. This aligns with linguistic relativity perspectives (Whorf 1956), which suggests that language structure influences cognition. Utilizing InSp, proficient individuals can effectively identify key features of a problem and suppress irrelevant details, thus organizing information hierarchically and reducing cognitive load during complex tasks (Fernyhough and Borghi 2023).

In addition, verbal labeling also helps structure concepts into categorical hierarchies (Alderson-Day and Fernyhough 2015), supporting movement between concrete instances and general principles (Lupyan 2009), which is a hallmark of CT fluency (Qian and Choi 2023). Research by Chi et al. (1989), further, demonstrates that verbal self-explanations facilitate the extraction of abstract principles from concrete examples, enabling schema transfer to novel problems. This finding supports the notion that language provides critical support for abstraction processes in CT.

Cognitive architecture models, curiously, indicate that InSp often serves as an *interface* between the sensory experience and abstract concepts in long-term memory (Morin 2005). This interface may be critical in CT tasks that require “translating” concrete problem details into abstract frameworks (Qian and Choi 2023). In practice, verbal self-guidance facilitates schema retrieval and manipulation in working memory, supporting a mental workspace for abstraction. This echoes Chella and Pipitone’s (2020) model of InSp as coordinating the phonological loop and episodic buffer. Moreover, InSp also supports mental model construction, which represents internal simulations that allow abstract solutions to be tested and refined before implementation (Borghi 2020). Building on this foundation, we now turn to algorithmic thinking, a CT process well aligned with the sequential nature of InSp.

### 3.4. InSp and Cognitive Processes in Algorithmic Thinking

The fourth cornerstone of CT, algorithmic thinking, whereby step-by-step procedures for solving problems are articulated, requires sequential reasoning and procedural planning (Lee et al. 2022). Empirical work appears to support this connection. For instance, when participants must silently rehearse an irrelevant word, their accuracy drops on sequential-planning tasks, indicating that covert verbal sequencing helps maintain the ordered action plan (Welsh and Huizinga 2005). It makes logical sense that InSp provides support for this CT process through several cognitive mechanisms related to sequential processing and mental simulation. The *temporal* and *progressive* nature of InSp makes it well-suited for step-by-step planning. Unlike visual imagery, which may represent multiple elements simultaneously, InSp unfolds sequentially (Fernyhough and Fradley 2005), mirroring the sequential nature of algorithms. This natural alignment between the sequential structure of InSp and algorithmic procedures provides a cognitive medium for developing solution sequences. 

When formulating algorithms, computational thinkers must consider logical relationships, particularly those involving contingencies, exemplified as “if–then” relationships (Lodi and Martini 2021). It would be logical to suggest that InSp supports this process through the verbal formulation of conditional statements. The propositional nature of language allows precise articulation of logical relationships (Fernyhough and Borghi 2023) that form the backbone of algorithmic thinking. By “talking through” these logical structures internally, computational thinkers can evaluate their coherence before implementation. Perhaps most significantly, it can be proposed that InSp enables mental simulation of algorithm execution. Through internal dialog, computational thinkers can mentally trace the execution of an algorithm, predicting outcomes and identifying potential issues. This simulation function aligns with the role of InSp in other planning domains, where verbal self-guidance supports the mental rehearsal of action sequences before execution. The *dialogic* quality of InSp (i.e., its conversation-like nature, all transpiring internally within an individual’s mind) supports adopting different perspectives on one’s own solution. This capacity for flexible *perspective-shifting* enables computational thinkers to view their algorithms from multiple viewpoints, essentially creating an internal dialog between “programmer” and “user” perspectives. This cognitive flexibility, it is contended, supports refinement and optimization of algorithms before implementation. 

### 3.5. InSp and (Meta)cognitive Processes in Debugging

Although traditionally not considered among the four cornerstones of CT (e.g., Wing 2006), debugging represents a critical metacognitive practice that drives the iterative refinement across CT components. As seen in Figure 1, rather than merely correcting algorithmic errors, debugging encompasses the continuous monitoring, evaluation, and revision of computational solutions. This iterative debugging process is particularly crucial during CT skill development, when learners must continuously refine their understanding through cycles of implementation, evaluation, and revision (Grover and Pea 2013; Zhang and Nouri 2019). InSp is vital to this process through several cognitive mechanisms related to error detection and hypothesis testing. Its dialogic nature supports hypothesis generation, such as “What could be causing this?” Such internal dialog helps generate possible explanations that can be systematically tested, as verbal formulation makes these hypotheses explicit, structured, and easier to evaluate. This metacognitive loop aligns with predictive-processing accounts, which frame cognition as the continuous minimization of prediction error (Clark 2013). In the VCS Model, dialogic InSp serves as the personal-level workspace where error signals are verbalized, inspected, and revised, thereby driving the debugging cycle across all CT operations. 

Verbal self-questioning also enhances metacognitive monitoring during debugging. Questions like “Does this make sense?” directed at oneself through InSp increase sensitivity to inconsistencies or unexpected outcomes. This heightened metacognitive awareness helps individuals detect subtle errors that might otherwise escape notice. Furthermore, the perspective-shifting capability of InSp enables individuals to simulate different viewpoints on their solution. By mentally adopting the perspective of an observer or user through internal dialog, they can identify assumptions or errors that remain invisible from their original perspective. This cognitive simulation of alternative viewpoints enhances error detection in ways that mirror collaborative debugging processes. When errors are detected, InSp supports flexible thinking by guiding individuals through different steps or strategies within their current problem-solving framework. Verbal self-guidance helps computational thinkers stay focused on the debugging process, sustaining effort despite setbacks. This ability to adapt and reorganize one’s approach in response to feedback is a key feature of CT, and it appears to rely heavily on InSp for planning, monitoring, and redirection.

The VCS Model acknowledges sub-personal theoretical frameworks such as predictive processing (Clark 2013; Friston 2010; Nave et al. 2022), which view cognition as continuous error minimization through predictive models and feedback loops. In CT, these mechanisms may enable early anomaly detection, while InSp provides the personal-level workspace for verbalizing, simulating, and manipulating solutions. This is particularly crucial in educational contexts where making cognitive processes visible supports instruction and transfer. Rather than competing explanations, sub-personal and personal-level accounts operate interactively. Predictive mechanisms initiate anomaly detection; then, InSp enables learners to articulate anomalies, generate hypotheses, and develop structured solutions. Debugging thus becomes not merely error-correction but an active process of cognitive articulation and organization, supporting adaptive CT in contexts prioritizing learner agency and reflective practice.

### 3.6. InSp as a Metacognitive Regulatory System

Beyond its specific role in each cornerstone and in debugging, InSp can seamlessly serves as a metacognitive regulatory system that coordinates across all components of CT. This overarching regulatory function supports executive processes that maintain goal-directed activity throughout complex problem solving. Through verbal formulation of goals and subgoals, InSp enhances their accessibility and precision in working memory (Chella and Pipitone 2020). These verbally mediated goal representations serve to help sustain focus on relevant aspects of complex problems over extended periods. 

Self-directed verbal commands also help direct attention to relevant problem features (Perrone-Bertolotti et al. 2014). Simple self-instructions like “work on this part first” should serve to help computational thinkers allocate and regulate cognitive resources efficiently during complex tasks. This “attentional control” utility becomes especially important when working with problems that involve multiple interacting components. Throughout the problem-solving process, InSp facilitates metacognitive monitoring through internal questioning (Pan et al. 2024). Questions directed at oneself such as “how am I progressing toward the goal?” support ongoing evaluation. Heaysman and Kramarski (2022) show that such self-monitoring prompts individuals to spot errors and impasses sooner and to verbalize alternative strategies. By articulating the advantages and disadvantages of different solution strategies as InSp, computational thinkers can make informed decisions about which approach to pursue. This adaptive strategy selection represents a sophisticated aspect of CT that relies heavily on verbal mediation. These regulatory functions echo cognitive-control models in which InSp maintains task context (Baddeley 2010) and supports self-regulation (Morin et al. 2018). The effectiveness of metacognitive-awareness interventions in CT education may, thus, stem from strengthening students’ capacity to externalize, then, internalize such verbal self-regulation (Pan et al. 2024).

### 3.7. Toward an Integrated Cognitive Model

Having examined how InSp supports each aspect of CT, from decomposition to algorithmic thinking and debugging, the relationship emerges as a complex, dynamic, and multifaceted system rather than a simple one-to-one mapping. The evidence suggests that InSp provides differentiated cognitive support, with specific functions selectively utilized for particular CT components. This nuanced relationship calls for an integrated model that captures both the synergistic operation of InSp functions and their differential contributions to computational cognition.

The cognitive mechanisms identified in previous sections operate not in isolation but as an integrated system wherein different InSp functions provide varying degrees of support for specific computational operations. Table 3 therefore extends Figure 1 and Table 1 and Table 2 by summarizing these predicted function-operation pairings in a single table.

Key patterns emerge: (a) sequential processing drives algorithmic thinking; (b) verbal working memory promotes decomposition; (c) symbolic representation supports both pattern recognition and abstraction; and (d) dialogic questioning with self-regulatory prompts powers debugging. The relative prominence of self-regulatory prompts across all CT components, especially debugging, positions metacognitive monitoring as both a general facilitator and the engine of iterative refinement. These alignments help refine earlier verbal-mediation theories by specifying precisely which language processes guide which computational operations (Huepe et al. 2011).

The matrix generates testable predictions; for example, articulatory suppression should impair decomposition more than pattern recognition; and blocking dialogic questioning should disproportionately affect debugging. These selective-interference designs transform the matrix into a research agenda. If specific InSp functions preferentially support particular CT components, then InSp profile variations should predict distinctive CT performance patterns, inviting investigation of how neurodivergent profiles achieve comparable outcomes through alternative routes, as pursued in Section 4.

### 3.8. Situating the VCS Model Within Theoretical Traditions

Having articulated the integrated cognitive architecture of the VCS Model, it is essential to position this framework within the broader theoretical landscape of cognitive science. Table 4 compares the VCS Model with five influential frameworks, showing both the continuities it preserves and the gaps it is designed to fill.

**Table 4:** Comparative Theoretical Architectures—Positioning the VCS Model. This table maps how the VCS model aligns with, and complements, established cognitive accounts related to InSp and CT (i.e., working memory, metacognitive control, sociocultural, executive function).

Theoretical Framework	Core Propositions	Primary Focus	Limitations Addressed by VCS Model vis-à-vis CT	Synergistic Integrations
**Working Memory Model** (Baddeley 1992, 2010; Cowan 1999; Oberauer 2002)	Phonological loop maintains verbal material via rehearsal (Baddeley) Central executive/attentional control allocates, shifts, and updates information (Baddeley; Cowan; Oberauer) Episodic buffer binds multimodal codes into integrated episodes (Baddeley) Working memory as the activated portion of long-term memory; a limited focus of attention (~4 chunks) constrains capacity (Cowan) A single-item “focus within focus” enables rapid updating (Oberauer)	Memory storage and manipulation mechanisms	Underspecifies role in complex reasoning Limited attention to metacognitive functions Minimal consideration of individual differences	VCS extends phonological loop beyond storage to active cognitive scaffolding, explicating how verbal rehearsal enables computational operations
**Metacognitive Control Theory** (Nelson and Narens 1990; Flavell 1979)	Monitoring processes track cognitive performance Control processes adjust strategies Metacognitive knowledge guides self-regulation	Self-awareness and strategic regulation	Undertheorizes specific mechanisms Limited integration with domain-specific reasoning Minimal attention to verbal mediation	VCS specifies InSp as primary metacognitive mechanism, detailing how verbal self-questioning enables debugging/“quality control”
**Vygotskian Sociocultural Theory** (Vygotsky 1962)	Language mediates higher psychological functions Development proceeds from social to individual plane Zone of proximal development guides learning	Developmental trajectory of verbal mediation	Broad developmental focus lacks computational specificity Limited attention to individual differences Underspecified cognitive mechanisms	VCS applies Vygotskian principles to computational domain, specifying mechanisms while acknowledging neurodivergent pathways
**Executive Function Frameworks** (Diamond 2013; Miyake et al. 2000)	Inhibition, shifting, updating as core components Domain-general cognitive control Individual differences in EF profiles	Cognitive control and flexibility	Minimal attention to verbal mediation Limited domain-specific applications Undertheorizes phenomenological aspects	VCS positions InSp as executive function mediator, explicating verbal contributions to computational flexibility
**CT Frameworks** (Wing 2006; Grover and Pea 2013) and the current gaps	CT as problem-solving approach Decomposition, abstraction, algorithms as core Transfer across domains	Training, educational and practical applications	Minimal cognitive mechanism specification Limited attention to individual differences Undertheorized psychological foundations	VCS provides cognitive architecture for CT, specifying mechanisms while maintaining educational relevance

Note. The working-memory row, Row 1, aggregates three complementary frameworks (i.e., Baddeley’s multicomponent model, Cowan’s embedded-processes view, and Oberauer’s focus-of-attention account), and they are all compatible with VCS as described. A detailed comparison is omitted as it lies beyond the article’s scope. The final row highlights that, in current CT frameworks, the underlying cognitive mechanisms are underexplored; it is included for context and is not presented as a cognitive theory.

Table 4 highlights both the compatibility and added value of the VCS Model relative to existing theories, particularly in clarifying underdefined mechanisms and integrating scattered constructs into a cohesive, testable framework. Collectively, these mappings clarify the functional architecture through which InSp supports CT, forming the conceptual basis for exploring alternative cognitive pathways in the following section. The following section briefly explores the theoretical possibility that non-verbal modalities (e.g., visual, motoric, or statistical processes) might also contribute to CT, although evidence remains limited and these alternative routes are not yet well understood.

## 4. Alternative Pathways to CT

The VCS Model, as discussed thus far, identifies InSp as the primary cognitive architecture supporting CT, notably in neurotypical individuals. However, the model is constructed to be non-exclusive in its theoretical stance. It recognizes that InSp, while predominant, may not necessarily be the only viable support through which CT is developed and engaged. Importantly, though, this pluralistic orientation is not intended to “flatten” all pathways into theoretical equivalence but, instead, to leave conceptual space for alternative mechanisms whose empirical and theoretical foundations are still emerging. This is because existing theoretical frameworks lend support to this possibility, although specific empirical evidence has been elusive.

Dual coding theory (Paivio 1990), embodied cognition models (Barsalou et al. 2003), and modality-specific reasoning accounts (Chrysikou et al. 2014) each suggest that complex cognitive operations can be guided through diverse representational systems (i.e., verbal, visual, spatial, or sensorimotor). While such systems have not yet been clearly mapped onto CT components or computational reasoning, they present important blueprints for future work, particularly in populations whose InSp profiles diverge from neurotypical patterns. A key population of interest, for instance, includes individuals who report a complete or near-absence of InSp, or anendophasia. Recent studies (Nedergaard and Lupyan 2024) suggest that these individuals may still demonstrate strong performance on tasks associated with CT, raising the possibility that non-verbal representational systems, such as visual imagery, spatial manipulation, or motor simulation, may offer alternative cognitive routes.

The proposed model here does not attempt to detail these pathways due to a general absence of inquiry into these aspects of computational reasoning, but it considers their plausibility to be a significant frontier for future empirical study. Additional support for alternative support mechanisms primarily arises from research on ASC. Individuals with ASC often show both atypical patterns of InSp usage and disproportionate strengths in computational domains such as programming (Muñoz et al. 2018). For example, Williams et al. (2012) found that some individuals with ASC relied on InSp for working memory maintenance but not for planning, indicating selective deployment. Rather than viewing this as a breakdown of verbal mediation, such findings may suggest adaptive strategies that leverage non-vernal (e.g., perceptual, detail-oriented, or rule-based) processing styles as effective supports for computational reasoning. Taken together, these results suggest a need to move beyond a rigid dichotomy of “verbal vs. non-verbal” pathways. Instead, the VCS Model adopts a modal framework: it identifies InSp as the most commonly observed and theoretically supported pathway but remains open to the possibility of multiple realizable routes to computational cognition. The absence of verbal scaffolding, based on previous research, does not necessarily appear to imply a deficit; rather, it may indicate the utility of alternative cognitive routes. It would therefore be reasonable to suggest, though not confirm at this time, that CT may arise through non-verbal means.

Future research should thus explore whether different profiles of InSp phenomenology predict distinct patterns of CT engagement, task preference, or problem-solving strategies. It is possible that visual thinkers, for example, decompose problems differently or represent algorithmic sequences through spatial metaphors. However, until such mechanisms are systematically specified, the current model remains focused on the best-evidenced route: verbal support through InSp. In sum, the VCS Model provides a theoretically flexible yet empirically grounded architecture for understanding the cognitive underpinnings of CT. It is offered not as a universal explanation but as a modal account, one that foregrounds InSp while remaining structurally open to expansion as the science of cognitive diversity advances.

Beyond verbal mediation, alternative cognitive architectures may support CT through non-linguistic modalities. Visual–spatial reasoning can underpin abstraction by enabling mental manipulation of spatial relationships and symbolic patterns without verbal encoding. Similarly, embodied cognition may facilitate debugging by linking action feedback and sensory prediction errors to problem correction, providing an iterative learning loop parallel to verbal reflection. Such routes illustrate that CT’s cognitive realization may be multimodal, with InSp representing one highly elaborated predominant pathway. Having established the theoretical flexibility of the VCS Model, the following section outlines how this architecture can be translated into testable hypotheses and empirical investigations.

## 5. From Theoretical Architecture to Empirical Investigation: Testing the VCS Model

The VCS Model advances a primarily verbal framework for understanding CT, grounded in well-documented mechanisms of InSp. Additionally, it remains open to the inclusion of non-verbal cognitive pathways, positioning itself as a platform for generating empirically testable hypotheses about both established, verbally based, and emerging, likely non-verbal routes to CT engagement and individual variation. If distinct cognitive architectures support CT through different mechanisms, interventions should be tailored to learners’ dominant cognitive pathways rather than assume universal benefit from verbally mediated strategies. This theoretical insight, it is alleged, would transforms how research and practice are envisioned and approached in CT development. For instance, experimental designs could manipulate verbal articulation (InSp engagement) during decomposition tasks to examine its causal contribution to CT performance. In such designs, participants might complete problem-solving tasks under articulatory suppression versus silent conditions, allowing direct assessment of InSp’s scaffolding role. Similar paradigms could test the hypothesized functions of verbal abstraction or hypothesis generation across CT components. Such paradigms would provide empirical traction for evaluating the model’s predicted mechanisms and their relation to individual differences in verbal–cognitive processing.

### 5.1. Implications for Cognitive Enhancement

For the majority of individuals, whose cognitive architecture privileges verbal mediation, targeted interventions leveraging InSp mechanisms would serve to enhance CT processes profoundly. Contemporary research in cognitive training supports this prediction, demonstrating that explicit instruction in verbal self-guidance enhances executive function across domains (Daches Cohen et al. 2023; Zelazo et al. 2018).

Three evidence-based intervention strategies may emerge from the VCS framework. First, metacognitive verbalization protocols involve structured exercises that translate implicit reasoning into explicit verbal sequences, making computational thinking processes conscious and refinable. Second, graduated internalization follows Vygotskian principles, systematically progressing from overt to covert verbal mediation as learners develop CT expertise. Third, domain-specific verbal schemas cultivate specialized InSp patterns optimized for particular computational operations—for instance, compressed verbal sequences for conditional logic in algorithmic thinking.

Crucially, the pluralistic approach also predicts that individuals with non-verbal cognitive architectures may benefit more from visual-spatial or embodied interventions. This necessitates assessment tools that identify dominant cognitive pathways before intervention design, ensuring that enhancement strategies align with rather than oppose natural cognitive tendencies.

### 5.2. Empirical Predictions and Hypotheses

The pluralistic nature of the proposed VCS Model generates clusters of empirically testable predictions that extend beyond simple correspondence between InSp and CT. By recognizing multiple cognitive architectures, the model produces differential predictions based on individuals’ dominant cognitive tendencies, creating opportunities for more nuanced empirical validation than traditional single-pathway models would permit. The following set of predictions, summarized in Table 5 at the end of this section, transform the theoretical framework into empirically testable hypotheses.

#### 5.2.1. Verbal Interference Effects: Testing the Primary Pathway

The model’s central claim that InSp serves as the primary support for CT yields specific predictions about potential interference effects. The following predictions regarding the centrality of InSp in CT align with recent findings by Borges and Mueller (2024), who document substantial individual differences in how InSp supports higher-order cognition. If verbal mediation indeed supports CT through the mechanisms outlined in Table 3, it can be posited that disrupting InSp should produce predictable patterns of impairment across CT components. Specifically, it may be predicted that articulatory suppression will selectively impair *algorithmic thinking* more than *pattern recognition* in neurotypical populations (H1a), reflecting the differential reliance on sequential verbal processing versus parallel visual-spatial mechanisms. Crucially, the pluralistic framework also predicts attenuated interference effects in populations with alternative cognitive architectures (H1b). Specifically, individuals with ASC or anendophasia, who may rely more heavily on visual-spatial pathways, should demonstrate resilience to verbal interference. This could serve to support the view that alternative cognitive architectures are to be treated, not as a deficit, but as alternative processing routes. This prediction transforms what may appear to be “null results” in traditional frameworks into positive evidence for cognitive plurality. The model further predicts a double dissociation (H1c); specifically, while neurotypical individuals should show greater vulnerability to verbal than visual–spatial interference, neurodivergent populations should exhibit the inverse pattern. This “crossover interaction” would provide compelling evidence for distinct cognitive architectures rather than simply impaired verbal processing.

#### 5.2.2. Developmental Trajectories: Emergence of Cognitive Pathways

The VCS Model makes specific predictions about how InSp–CT relationships emerge across development. Rather than assuming fixed relationships, it may be predicted that the relationship between InSp efficiency and CT performance strengthens during middle childhood (H2a), coinciding with the consolidation of verbal mediation strategies and the increasing complexity of computational reasoning demands. Perhaps more provocatively, the model predicts that early sensory preferences and processing styles may forecast later pathway utilization (H2b). Children who demonstrate early preferences for visual–spatial over verbal–sequential processing may be more likely to develop non-verbal CT strategies, suggesting that alternative pathways represent primary architectural differences rather than, necessarily, compensatory mechanisms developed in response to verbal processing difficulties.

#### 5.2.3. Neural Mechanisms: Biological Substrates of Cognitive Plurality

The VCS Model’s pluralistic approach generates distinct neurobiological predictions. For individuals utilizing the verbal pathway, the model would predict activation of classic language networks during CT tasks, particularly, left inferior frontal regions associated with InSp (H3a). This activation should be most pronounced during *algorithmic thinking* and *debugging* phases, consistent with the functional mappings in Table 3. Conversely, individuals utilizing alternative pathways would demonstrate enhanced activation in visual–spatial networks during the same CT tasks (H3b). Importantly, both patterns should correlate with successful task performance, providing neurobiological evidence for multiple valid routes to CT proficiency. The absence of typical language network activation in successful problem solvers would not indicate dysfunction but, rather, the operation of an alternative cognitive architecture, according to the proposed model. 

#### 5.2.4. Educational Applications: Matching Interventions to Cognitive Tendencies

Perhaps most significantly for practice, the VCS Model predicts aptitude–treatment interactions in CT instruction and training. Verbal mediation training should prove more effective than visual–spatial training for most neurotypical learners (H4a), while the inverse pattern should hold for individuals with non-verbal cognitive tendencies (H4b). This interaction effect would validate the importance of assessing cognitive profiles before implementing interventions, moving beyond one-size-fits-all approaches to CT education. For instance, the model predicts that teaching *algorithmic thinking* through verbal self-explanation protocols will differentially benefit learners based on their InSp profiles, while visual flowcharting methods may prove more effective for those with minimal InSp experience.

The possible empirical explorations outlined in Table 5 demands methodological sophistication to align with the complexity of the VCS Model. The multidimensional nature of both InSp and CT, combined with the recognition of multiple cognitive pathways, would require a departure from conventional univariate experimental approaches. The following sections articulate methodological frameworks suitable for capturing the cognitive plurality while maintaining the precision required for hypothesis testing.

## 6. Implications and Future Research Directions

The VCS Model introduced in this manuscript takes am inclusive approach that has meaningful implications across several fields and opens the door to new lines of empirical research. By framing the verbal link between InSp and CT as a primary, but not exclusive, pathway, the model shifts our understanding of computational cognition, educational practice, and cognitive diversity from a singular view to a more flexible, inclusive framework.

### 6.1. Theoretical Implications

The VCS Model advances cognitive science vis-à-vis InSp and CT in several key directions. First, it expands working memory theory beyond storage functions to active cognitive scaffolding, demonstrating how the phonological loop enables computational operations through specific mechanisms (see Table 2). This conceptualization suggests that verbal working memory contributes to complex reasoning in ways that current models “underspecify.” Second, the proposed framework provides a mechanistic account of how metacognitive processes operate in CT contexts. By positioning InSp as the primary vehicle for metacognitive monitoring in CT, the model specifies how abstract self-regulatory processes manifest in concrete cognitive operations. This specificity advances metacognitive theory from broad principles to testable mechanisms.

Third, the nuanced nature of the VCS Model challenges and complements monolithic theories of language–thought relationships. Rather than asking *whether* language shapes thought, the VCS Model aims to identify *how* verbal mediation represents one optimization strategy among several alternative possibilities for achieving specific aspects of CT reasoning. This multiplicity enriches debates about linguistic relativity by showing how language can support cognition without determining it.

### 6.2. Educational Implications

The recognition of multiple pathways to CT proficiency demands fundamental reconceptualization of educational approaches. Rather than assuming learners benefit from verbal scaffolding strategies, as much of the existing research presupposes (Zhang and Nouri 2019), educators should first assess learners’ dominant cognitive pathways. The empirical predictions outlined in Table 5 suggest concrete assessment protocols: learners who show resilience to articulatory suppression during CT tasks likely utilize alternative pathways and may benefit more from visual-spatial or embodied instructional approaches.

This cognitive profiling approach extends beyond simple learning style preferences to identify fundamental differences in cognitive architecture. For neurotypical learners with strong InSp, interventions should leverage verbal self-explanation protocols, metacognitive questioning strategies, and graduated internalization from overt to covert speech. For learners with minimal InSp or neurodivergent profiles, instruction should emphasize visual pattern mapping, spatial manipulation of abstract structures, and embodied problem-solving approaches.

### 6.3. Future Research Priorities

Section 5 presented a set of focused, testable hypotheses aimed at empirically evaluating the VCS Model in relation to existing theoretical and empirical work. Building on that foundation, and as summarized in Table 5, this section broadens the research agenda to include complementary and exploratory directions. These include methodological innovation, developmental trajectories, cross-cultural variation, and intervention design.

#### 6.3.1. Mechanistic Studies

While Section 5 emphasized interference paradigms as a tool for mapping InSp–CT dependencies, future research should also explore convergent, real-time methodologies that capture dynamic cognitive engagement. Eye-tracking during CT tasks could reveal whether verbal versus visual processors show different scanning patterns. Pupillometry might distinguish cognitive load profiles across pathways. Also, think-aloud protocols, analyzed for linguistic complexity and structure, could illuminate how verbal scaffolding operates in real-time. In addition to identifying dominant pathways across individuals, finally, future research should also investigate whether cognitive strategies shift within across situations due to such factors as task characteristics. It may be conceivable that, for example, the same individuals may rely heavily on verbal mediation for planning tasks but, say, adopt visual-spatial strategies for pattern recognition or debugging. Understanding these within-person variations could clarify how cognitive flexibility contributes to CT proficiency, and may also inform the development of adaptive instructional and assessment tools.

#### 6.3.2. Developmental Investigations

Building on the developmental hypotheses in Section 5, future research should pursue longitudinal and cross-cultural studies to examine how linguistic environments and early interventions shape the evolving relationship between InSp and CT over time. Longitudinal studies tracking both InSp “sophistication” and CT development could test whether their correlation strengthens throughout learner development. Of particular interest may be whether early interventions targeting specific InSp functions (e.g., dialogic self-questioning) produce trickledown improvements in *debugging* abilities, for example. Cross-cultural comparisons could examine whether languages with different structural properties produce variations in InSp-CT relationships.

#### 6.3.3. Neurodivergent Populations

Section 5 discussed cognitive architecture differences through the lens of interference and neural activation; in addition, further studies are needed to probe neurodivergent populations as windows into alternative CT pathways and even potential biomarkers. The model’s boundary conditions call for systematic investigation. How do individuals with developmental language disorders navigate CT tasks? Do those with exceptional visual–spatial abilities (but typical language skills) show distinct neural activation patterns during computational reasoning? Can we identify biomarkers that predict which cognitive pathway will be dominant? Such investigations would not merely demonstrate differences but, instead, may reveal the fundamental flexibility of human computational cognition. By studying populations who achieve CT proficiency through alternative routes, we could gain insight into our capacity for multiple solutions to complex cognitive challenges, ultimately enriching our understanding of cognitive architecture itself.

#### 6.3.4. Intervention Studies

The aptitude–treatment interactions predicted by the VCS Model call for sophisticated experimental designs that go beyond simple comparisons of verbal versus visual instructional formats. Future research should explore how to optimize CT instruction for learners across the InSp continuum. For example, can adaptive learning environments assess cognitive profiles in real time and adjust scaffolding strategies accordingly? Interventions might include guided self-questioning prompts, structured verbal rehearsal tools, or instructional routines designed to strengthen specific InSp functions such as planning or error monitoring. In parallel, it would be important to examine how these interventions impact both CT outcomes and broader metacognitive development. By designing interventions aligned with the learner’s dominant cognitive pathway, researchers can test the model’s claims while contributing to more inclusive and effective instructional practices. These efforts would not only advance theoretical validation of the VCS Model but also support real-world applications in education and cognitive training.

#### 6.3.5. Technological Applications

Another promising possibility for future research involves the design and empirical testing of CT tools informed by the VCS Model. Specifically, researchers could investigate how programming environments might be tailored to align with different cognitive pathways. For learners who rely on verbal mediation, design features might include embedded prompts for self-explanation, interactive debugging questions that mimic dialogic InSp, or visualizations of verbal reasoning chains. Conversely, for learners utilizing alternative cognitive pathways, research could explore the effectiveness of tools that emphasize direct manipulation, visual pattern libraries, or spatial program representations. These investigations could clarify how adaptive technological environments support CT development across diverse cognitive profiles and contribute to more inclusive instructional design.

The research potential outlined here, along with the broader empirical and theoretical priorities outlined thus far in this manuscript, point toward an exciting range of research possibilities, each with potential for far-reaching implications beyond InSp–CT relationship.

## 7. Conclusions

This exploration has proposed the Verbal–Cognitive Scaffold (VCS) Model as a theoretical framework for understanding the cognitive mechanisms underlying computational thinking (CT), with particular emphasis on inner speech (InSp) as the predominant support process. Through systematic theoretical synthesis, the model explains how the multidimensional functions of InSp enable and structure the core operations of computational reasoning in most neurotypical populations. Given the limited attention to cognitive mechanisms in existing CT research, the VCS Model provides a much needed framework that incorporates both verbal and non-verbal pathways. It shifts the focus from a single, uniform pathway to a more inclusive account that recognizes cognitive variability. In this way, it opens new directions for understanding both typical and atypical cognitive characteristics and development, differentiated instructional design, and the underlying architecture of abstract reasoning, development, differentiated instructional design, and the broader architecture of abstract reasoning. The VCS Model’s primary contribution lies not in empirical demonstration but in theoretical synthesis, connecting previously disparate cognitive domains through precise mechanistic specification. This theoretical work serves the essential scientific function of generating novel hypotheses and research programs, following established traditions in cognitive science where conceptual frameworks precede and guide empirical investigation.

The proposed VCS Model and its empirical potential directly address the central guiding question and its subcomponents, which guided the present theoretical inquiry. To explore the specific cognitive support that InSp provides for CT, Section 3 offered detailed analyses of the mechanisms linking InSp to distinct CT components (Guiding Question 1), as summarized in Table 2. That section also identified the InSp functions most closely aligned with core CT operations (Guiding Question 2), as outlined in Table 3. Guiding Question 3, which examined the possibility of alternative non-verbal pathways, was the focus of Section 4. Finally, Section 6 considered the model’s intervention potential (Guiding Question 4), drawing on the empirical directions outlined in Section 5. Collectively, this theoretical analysis not only clarifies the cognitive architecture underlying CT but also lays the groundwork for future intervention design, including applications for neurodivergent learners, thus offering a comprehensive response to the central guiding question and its subcomponents.

The VCS Model makes several key contributions to cognitive science. To start, it offers the first known systematic mapping of how specific functions of InSp support distinct components of CT, moving beyond general presupposition about verbal mediation to identify concrete cognitive mechanisms. Second, the comprehensive nature of the model prompts experimentally testable hypotheses and predictions that differentiate between cognitive architectures rather than treat variation as aberrations. Third, it offers a framework for potentially understanding why certain populations excel at computing tasks despite *atypical* language processing, transforming apparent paradoxes into theoretical insights. Perhaps most significantly, the VCS Model demonstrates how theoretical frameworks in cognitive science can embrace complexity without necessarily sacrificing precision. By acknowledging multiple pathways while specifying their characteristics, the model provides tools for both researchers seeking to understand computational cognition and practitioners seeking to enhance it.

As discussed throughout, this proposed model should be empirically examined, and the limits and variability of its underlying mechanisms warrant further exploration. Even so, the framework offers a meaningful theoretical contribution. By proposing specific, testable links between previously unconnected cognitive domains explicitly, the VCS Model offers new tools for understanding how people develop and apply computational reasoning. In this way, the framework contributes to the larger goals of cognitive science, perhaps, to map the diverse and complex architecture of human thought.

## Figures and Tables

**Table 1:** Core Components of the Verbal–Cognitive Scaffold Model. Panel A lists the key dimensions of InSp and their cognitive functions relevant to CT. Panel B defines the four CT components, with debugging identified as a metacognitive overlay spanning them. Terminology corresponds to Figure 1, and Table 2 maps these functions to underlying mechanisms and CT components.

**Panel A: Phenomenological Dimensions of Inner Speech (InSp) Relevant to Computational Thinking (CT)**			
**Dimension**	**Description**	**Functional Mechanism**	**Relevance to CT**
Dialogicity	Conversational vs. monologic forms; internal Q&A sequence	Supports perspective-shifting, self-questioning, and hypothesis generation	Supports hypothesis testing and debugging through internal dialogue
Condensation	Syntactic compression relative to external speech	Enhances processing efficiency	Allows rapid cycling through task-relevant representations
Intentionality	Volitional vs. spontaneous emergence	Supports goal-directed thinking	Enables deliberate strategy selection and planning
Phenomenological Richness	Ranges from abstract to quasi-perceptual experience	Varies representational flexibility	Accommodates different levels of abstraction in problem-solving
**Panel B: Core Components of Computational Thinking (CT)**			
**Component**	**Definition**	**Key Cognitive Demands**	**Primary Operations**
Decomposition	Breaking complex problems into manageable parts	Hierarchical representation, chunking	Problem division, subgoal identification
Pattern Recognition	Identifying similarities within/between problems	Categorization, analogical reasoning, schema activation/analogical mapping	Feature extraction, similarity matching
Abstraction	Focusing on relevant information while filtering details	Selective attention, generalization, concept formation	Essential feature isolation, principle extraction
Algorithmic Thinking	Articulating step-by-step solution procedures	Sequential planning, conditional logic	Procedure specification, logical sequencing
Debugging	Iterative error detection and correction (metacognitive overlay) as needed	Error monitoring, hypothesis generation	Solution testing, strategy revision

**Table 2:** Mapping InSp functions to CT components via Cognitive Mechanisms. This table maps InSp functions to the cognitive mechanisms that support each CT cornerstone and operationalizes Figure 1, including debugging as an as-needed metacognitive overlay. The mapping is theoretically derived and offered as testable predictions; potential measures are included to guide empirical work.

CT Processes	InSp Function	Cognitive Mechanism	How It Works	Example	Potential Measurement Approaches
**Decomposition**	Verbal articulation	Working memory support through phonological loop	InSp maintains problem components in active memory while analyzing relationships	*Planning a family vacation by mentally saying “We need to handle transportation, accommodations, activities, and budget,” rather than trying to solve it as one overwhelming problem*	Articulatory suppression paradigm Working memory span tests Think-aloud protocols during decomposition tasks
**Pattern** **Recognition**	Verbal labeling	Categorization and memory retrieval	Verbal labels create retrievable categories that highlight structural similarities	*Noticing similarities in different math problems and thinking, “These all involve finding the area of different shapes, just like we did last week.”* *Identifying recurring patterns within a math problem and thinking, “This problem involves a sequence of repetitive calculations, so I can use a short cut.”*	Categorization speed/accuracy with/without verbal interference Assessment of domain-specific labeling vocabulary
**Abstraction**	Symbolic representation	Generation of abstract concepts through linguistic labels	Language provides symbols that can represent abstract relationships independent of specific instances	*Creating a mental concept of “exam preparation” that applies to any subject, focusing on common elements (reviewing, practicing, checking) while filtering out subject-specific details*	Abstract concept formation tasks Measurement of abstraction level in problem descriptions
**Algorithm** **Design**	Sequential processing	Step-by-step planning through temporal unfolding	The linear nature of InSp naturally structures procedural sequences	*Mentally walking through a cooking recipe: “First preheat the oven, then mix the ingredients, next pour into a pan, finally bake for 30 min.”*	Sequential planning assessments Algorithm complexity analysis with/without verbal interference
**Debugging**	Dialogic self-questioning; Self-regulatory prompts	Metacognitive monitoring; hypothesis generation	Internal Q&A format enables systematic error checking and solution testing	*When a cake doesn’t rise properly, asking oneself “Did I forget the baking powder? Or is the oven broken?”*	Metacognitive awareness inventories Error detection efficiency measures Self-regulation assessments

**Table 3:** Theoretical Matrix of InSp-CT Functional Relationships. Theoretical predictions from cognitive mechanism analysis, linking InSp functions with specific aspects of CT. Helps generate testable hypotheses presented in Table 4.

INNER SPEECH FUNCTION	DECOMPOSITION	PATTERN RECOGNITION	ABSTRACTION	ALGORITHMIC THINKING	DEBUGGING
**SEQUENTIAL PROCESSING**	Moderate	Low	Low	**HIGH**	Moderate
**VERBAL WORKING MEMORY**	**HIGH**	Moderate	Moderate	Moderate	Low
**SYMBOLIC REPRESENTATION**	Low	**HIGH**	**HIGH**	Low	Low
**DIALOGIC QUESTIONING**	Moderate	Low	Moderate	Moderate	**HIGH**
**SELF-REGULATORY PROMPTS**	Moderate	Moderate	Moderate	Moderate	**HIGH**

**Table 5:** Empirical Predictions of the Pluralistic VCS Model. Testable hypotheses derived from the VCS Model across four empirical domains. Each hypothesis set contrasting predictions for individuals utilizing verbally mediated versus alternative cognitive pathways, testing the proposed multiple routes to CT.

Hypothesis Set	Label	Prediction	Theoretical Rationale	Methodological Approach
**Verbal Interference Effects**	H1a	Articulatory suppression selectively impairs algorithmic thinking > pattern recognition (neurotypical)	Algorithmic thinking requires sequential verbal processing; pattern recognition utilizes parallel visual-spatial mechanisms	Dual-task paradigm with computational thinking tasks
	H1b	Attenuated interference effects in ASC/anendophasia populations	Alternative cognitive pathways confer resilience to verbal disruption	Comparative study: neurotypical vs. neurodivergent
	H1c	Visual-spatial interference shows inverse pattern in neurodivergent populations	Reliance on visual-spatial pathways creates vulnerability to non-verbal disruption	Double dissociation design
**Developmental** **Trajectories**	H2a	InSp sophistication × CT performance correlation strengthens during middle childhood	Functional interdependence emerges during critical developmental period	Longitudinal cohort study
	H2b	Early sensory preferences predict alternative pathway utilization	Cognitive architectures manifest early; not merely compensatory	Prospective developmental tracking
**Neural Mechanisms**	H3a	Left inferior frontal activation during CT tasks (verbal mediators)	InSp networks recruited for computational operations	fMRI during CT tasks
	H3b	Enhanced visual-spatial network activation (alternative pathway users)	Distinct neural substrates for non-verbal CT	fMRI with cognitive profiling
**Educational** **Applications**	H4a	Verbal mediation training > visual-spatial training (neurotypical)	Intervention alignment with dominant cognitive architecture	Randomized controlled trial
	H4b	Inverse training effectiveness pattern (non-verbal preference)	Cognitive profile- intervention matching optimizes outcomes	Aptitude-treatment interaction design

## Data Availability

No new data were created or analyzed in this study. Therefore, data sharing is not applicable.
